# Prediction of Large Joint Destruction in Patients With Rheumatoid Arthritis Using ^18^F-FDG PET/CT and Disease Activity Score

**DOI:** 10.1097/MD.0000000000002841

**Published:** 2016-02-18

**Authors:** Takahito Suto, Koichi Okamura, Yukio Yonemoto, Chisa Okura, Yoshito Tsushima, Kenji Takagishi

**Affiliations:** From the Department of Orthopaedic Surgery (TS, KO, YY, CO, KT), Gunma University Graduate School of Medicine, Showamachi, Maebashi, Gunma, Japan ; Department of Orthopaedics (KO), The Warren Alpert Medical School of Brown University/Rhode Island Hospital, Providence, RI ; and Department of Diagnostic Radiology and Nuclear Medicine (YT), Gunma University Graduate School of Medicine, Showamachi, Maebashi, Gunma, Japan.

## Abstract

The assessments of joint damage in patients with rheumatoid arthritis (RA) are mainly restricted to small joints in the hands and feet. However, the development of arthritis in RA patients often involves the large joints, such as the shoulder, elbow, hip, knee, and ankle. Few studies have been reported regarding the degree of large joint destruction in RA patients. ^18^F-fluorodeoxyglucose positron emission tomography combined with computed tomography (FDG-PET/CT) visualizes the disease activity in large joints affected by RA. In this study, the associations between destruction of the large joints and the findings of FDG-PET/CT as well as laboratory parameters were investigated, and factors associated with large joint destruction after the administration of biological therapy were identified in RA patients.

A total of 264 large joints in 23 RA patients (6 men and 17 women; mean age of 66.9 ± 7.9 years) were assessed in this study. FDG-PET/CT was performed at baseline and 6 months after the initiation of biological therapy. The extent of FDG uptake in large joints (shoulder, elbow, wrist, hip, knee, and ankle) was analyzed using the maximum standardized uptake value (SUVmax). Radiographs of the 12 large joints per patient obtained at baseline and after 2 years were assessed according to Larsen's method. A logistic regression analysis was performed to determine the factors most significantly contributing to the progression of joint destruction within 2 years.

Radiographic progression of joint destruction was detected in 33 joints. The SUVmax at baseline and 6 months, and the disease activity score (DAS) 28-erythrocyte sedimentation rate (ESR) at 6, 12, and 24 months were significantly higher in the group with progressive joint destruction. The SUVmax at baseline and DAS28-ESR at 6 months were found to be factors associated with joint destruction at 2 years (*P* < 0.05).

The FDG uptake in the joints with destruction was higher than that observed in the joints without destruction. The SUVmax at baseline and the DAS28-ESR at 6 months after the biological treatment were identified to be significant factors predicting destruction of the large joints at 2 years.

## INTRODUCTION

Rheumatoid arthritis (RA) is a systemic, inflammatory autoimmune disorder characterized by chronic inflammation of the joints and bone destruction. The recent development of biologics, such as tumor necrotic factor (TNF)-blocking agents, for the treatment of RA has improved the outcomes of the disease, and remission remains the ideal aim in patients with RA.^1^ Obtaining a prompt and accurate diagnosis and providing early aggressive treatment using biologics are keys to achieving effective management in cases of RA.^2^ As increased joint damage may cause functional impairments, it is important to inhibit radiographic progression and predict consequent joint destruction.

Recent investigations have shown that power Doppler ultrasonography (PDUS) is useful for predicting joint destruction of the hands in RA patients.^3–5^ PDUS detects the synovial perfusion in the inflamed joints, and the synovial perfusion detected on PDUS is related to subsequent radiographic progression.^4^ Although these assessments of joint damage are mainly restricted to small joints in the hands and feet, the development of arthritis in RA patients often involves the large joints, such as the shoulder, elbow, hip, knee and ankle, in addition to the fingers.^6^ As radiographic damage, the damage that is radiographically detectable, of large weight-bearing joints is strongly associated with a disability in walking and functions as an important determinant of the functional capacity in patients with RA,^7,8^ it is indispensable to assess the extent of radiographic damage in these joints. However, few previous reports have studied the predictive value of radiographic findings for destruction of the large joints in RA patients.

^18^F-Fluorodeoxyglucose (FDG)-positron emission tomography (PET) imaging may be used to assess the metabolic activity of synovitis directly and measure the disease activity in large joints affected by RA.^6,9–11^ Whole-body FDG-PET combined with computed tomography (CT) (FDG-PET/CT) is able to visualize the disease activity in large joints affected by RA.^6^ Furthermore, the response on FDG-PET correlates with the clinical response to biologic treatment in cases of RA.^12–14^ However, it is not thoroughly understood whether ^18^F-FDG-PET/CT findings correlate with the severity of destruction in the large joints of the RA patients. The purpose of this study was therefore to investigate the associations between destruction of the large joints and ^18^F-FDG PET/CT findings, the disease activity, and laboratory parameters in RA patients.

## MATERIALS AND METHODS

### Subjects

The Institutional Review Board of our hospital approved the protocol for this study. Between May 2010 and November 2012, 23 patients (6 men, 17 women; mean age 66.9 ± 7.9 years) were enrolled. Based on the power analysis with G^∗^Power 3 (Faul, Erdfelder, Lang& Buchner, 2007: http://www.psycho.uni-duesseldorf.de/abteilungen/aap/gpower3/*),* the sample size of this study was calculated enough to supply 80% power. All patients were diagnosed according to the American College of Rheumatology criteria revised in 1987. Informed consent was obtained from all subjects before participation in this study. Most patients had been previously treated with disease-modifying antirheumatic drugs (DMARDs), such as methotrexate (MTX) and prednisolone (PSL), and 6 patients had received anti-TNF agents, including infliximab (IFX) in 3 patients, adalimumab (ADA) in 2 patients, and etanercept (ETN) in 1 patient. Based on the clinically inadequate responses to these previous treatments, the patients were recommended to receive treatment with biologics, such as anti-TNF agents and anti-interleukin-6 receptor antibodies. Whole-body ^18^F-FDG PET/CT was performed at baseline, after which the patients were administered biological therapy, and again at 6 months after the start of treatment. Radiographs of the large joints were obtained at baseline and 2 years after the initiation of therapy.

Clinical parameters, including the erythrocyte sedimentation rate (ESR) and levels of C-reactive protein (CRP), matrix metalloproteinase-3 (MMP-3), anticyclic citrullinated peptide antibodies (ACPA), and rheumatoid factor (RF), were also assessed at baseline and 6, 12, and 24 months after the initiation of biological therapy. The disease activity was evaluated at the same time using the disease activity score in 28 joints (DAS28), DAS28-CRP, simplified disease activity index (SDAI), and clinical disease activity index (CDAI).

### PET Imaging

After >6 hours of fasting, whole-body PET was completed following the intravenous injection of ^18^F-FDG (5 MBq/kg). One hour after ^18^F-FDG injection, the acquired data were conducted in 3-dimensional mode using a PET-CT scanner (Biograph 16; Siemens Medical Solutions Inc, Munich, Germany). The patients were placed in a supine position and scanned from the head to the toe in arms-down position. Attenuation correction of the PET images was done using CT and followed by reconstruction with an ordered subsets expectation-maximization algorithm into 128 × 128 matrices. The nuclear physicians with >15 years’ experience interpreted the PET images. An increased FDG uptake in the bilateral shoulder, elbow, wrist, hip, knee, and ankle joints was recorded as described below according to previous reports.^12,13^ The standardized uptake value (SUV) was calculated for the semiquantitative assessment by attenuation-corrected transaxial images, the injected dose of ^18^F-FDG, patient's body weight, and cross-calibration factor between the PET images and dose calibrator as follows:

SUV = radioactive concentration in the region of interest (ROI) (MBq/g)/injected ^18^F-FDG dose (MBq)/patient's body weight (g).

At first the upper limit SUV was set as 5, the SUV images were observed. Then ROIs were manually drawn for each joint by a nuclear physician with the assistance of corresponding CT scans. If there was high FDG uptake over SUVmax > 5, the upper limit SUV was set as 8 or 10. To assess of the FDG uptake, the maximum SUV (SUVmax) in the ROI was considered as a representative value of the FDG uptake.

### Joint Destruction Assessment

Standard anteroposterior plain radiographs of the large joints (shoulder, elbow, wrist, hip, knee, and ankle) were used. The hip and ankle joints were exposed in the supine position, and the knee joints were exposed under conditions of weight bearing. Radiographs of the large joints were taken at baseline and 2 years later. Joints that had previously been treated with joint replacement surgery at baseline were excluded from the radiographic assessments. A history of total joint replacement within 2 years was regarded as being indicative of joint destruction progression. Two certified rheumatologists (K.O. and Y.Y.) assessed the degree of joint damage in consultation with each other according to the method of Larsen et al^15^ using standard reference films, without any information regarding the patients.

### Statistical Analysis

The statistical analyses were conducted using the SPSS Statistics 22 statistical analysis software program. The data are reported as the mean ± standard deviation (SD). Group differences in disease duration, baseline disease characteristics (ESR, CRP, MMP-3, ACPA, and RF), disease activity (DAS28, DAS28-CRP, SDAI, and CDAI) and SUV were analyzed using the Mann–Whitney *U* test. A logistic regression analysis was performed to determine the factors most significantly contributing to the progression of joint destruction within 2 years. *P* values of < 0.05 were considered to be statistically significant.

## RESULTS

The main clinical and laboratory characteristics are provided in Table 1. The mean age of the patients was 66.9 years, and the mean duration of RA was 13.8 years. IFX was used in 4 patients, ETN was used in 4 patients, and ADA was used in 6 patients. Tocilizumab (TCZ) was used in 9 patients, including 3 patients switched from IFX, 2 patients switched from ADA, and 1 patient switched from ETN due to side effects or a clinically inadequate response to the previous treatment. We assessed 12 large joints (bilateral joints of the shoulder, elbow, wrist, hip, knee, and ankle) per patient, for a total of 276 joints. Twelve joints had previously been treated with joint replacement surgery at baseline and were excluded from the analysis. Therefore, we evaluated 264 large joints in this study.

**TABLE 1:**
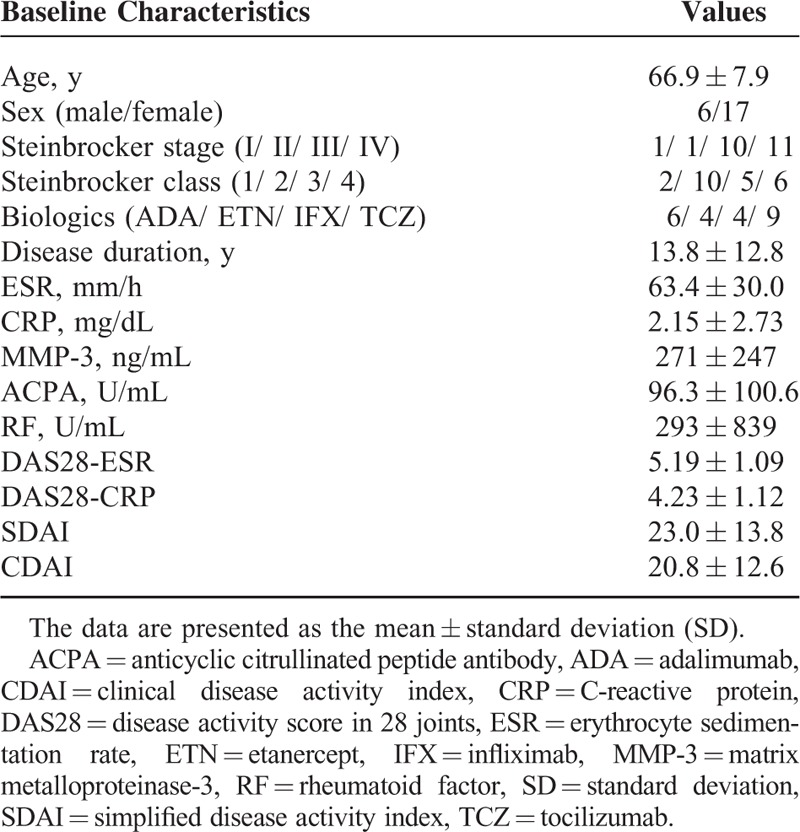
Baseline Demographic, Clinical, and Laboratory Features of the 23 Patients

Among the 264 joints in the patients with RA, radiographic progression of joint destruction was detected in 33 large joints (shoulder/elbow/wrist/hip/knee/ankle: 7/1/1/4/12/8), including 4 joints treated with surgery.

**FIGURE 1:**
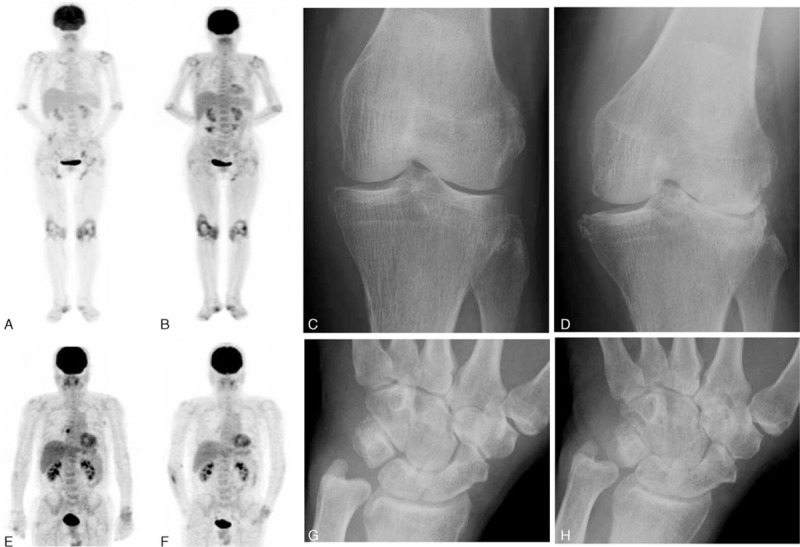
Typical ^18^F-FDG PET/CT images and radiographs with a progression of joint destruction. A 63-year-old woman with a 32-year history of RA treated with TCZ therapy. Anterior image obtained on ^18^F-FDG PET/CT at baseline (A) and 6 months after the initiation of biological therapy (B). The Larsen grade in the left knee joint progressed from grade III (C) to grade IV (D). A 70-year-old man with a 3-year history of RA treated with IFX therapy. ^18^F-FDG PET/CT findings at baseline and 6 months after treatment (E, F). The Larsen grade in the left wrist progressed from grade II to grade III (G, H). Progression of joint destruction was shown in the joints with high FDG uptake at baseline and 6 months. FDG-PET/CT = fluorodeoxyglucose positron emission tomography combined with computed tomography, RA = rheumatoid arthritis, TCZ = tocilizumab.

Figure 1 shows typical ^18^F-FDG PET/CT images of large joints exhibiting arthritis in RA patients. A 63-year-old woman with a 32-year history of RA received TCZ therapy, and her left knee joint subsequently demonstrated progression of joint damage within 2 years (Larsen grade change from III to IV). The SUVmax in the left knee was 5.19 at baseline and 3.36 at 6 months (Figure 1A–D).

A 70-year-old woman with a 3-year history of RA was treated with IFX therapy. Her grade II left wrist showed progression to grade III; the SUVmax in the left wrist was 1.73 at baseline and 2.54 at 6 months (Figure 1E–H).

The background characteristics of the patients with and without radiographic progression >2 years were evaluated. The SUV values at baseline and 6 months and the ΔSUV, the difference in the SUVmax of the joints before and after treatment, were also assessed between these 2 groups (Table 2).

**TABLE 2:**
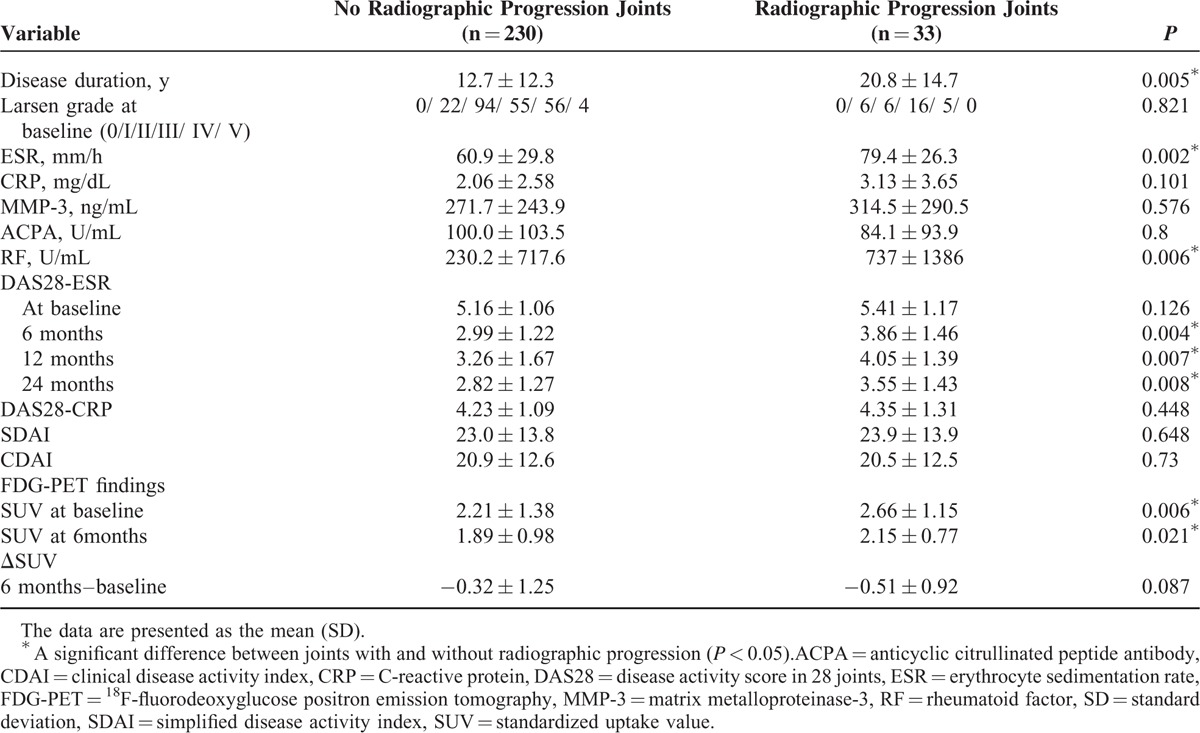
Parameters Contributing to the Radiographic Progression of Large Joint Destruction

There were no significant differences between the groups with and without radiographic progression with respect to the following characteristics: laboratory values at baseline (CRP, MMP-3, ACPA), DAS28-ESR at baseline, DAS28-CRP, SDAI, and CDAI. The disease duration, ESR level, and RF level at baseline were significantly higher in the group with radiographic progression of joint damage (*P* = 0.05, *P* = 0.002, and *P* = 0.006). Furthermore, the DAS28-ESR scores at 6, 12, and 24 months were also significantly higher in the group with progression of joint destruction (*P* = 0.006, *P* = 0.007, and *P* = 0.008).

**FIGURE 2:**
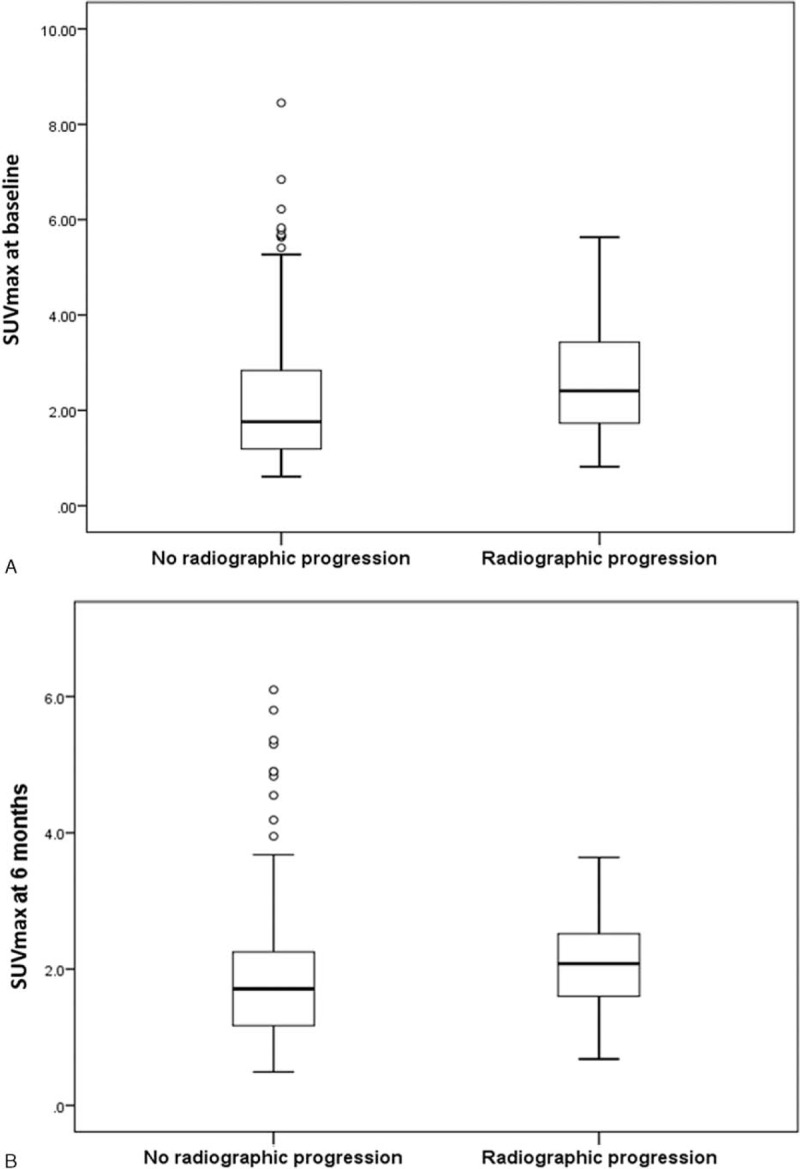
SUVmax values in the joints with and without radiographic progression. (A) SUVmax values at baseline. The SUVmax values were significantly higher in the joints with radiographic progression than in the joints without radiographic progression (*P* = 0.006). (B) SUVmax values at 6 months. The SUVmax values were significantly higher in the joints with radiographic progression than in the joints without radiographic progression (*P* = 0.021). SUVmax = maximum standardized uptake value.

Regarding the ^18^F-FDG PET/CT findings, the statistical analyses revealed that the SUVmax values at baseline and 6 months were significantly higher in the group with progressive joint destruction (*P* = 0.006 and *P* = 0.021) (Figure 2). As for the ΔSUVs, which represent clinical improvements in the affected joints over 6 months, there were no significant differences between the patients with radiographic progression of joint destruction at 2 years and those without progression.

The disease duration, DAS28-ESR scores at 6, 12, and 24 months, SUVmax values at baseline and 6 months, which were found to be significant variables in the univariate analyses, as well as age and sex were entered simultaneously into a stepwise multivariate logistic regression analysis to identify factors with independent predictive value for joint destruction within 2 years. The analysis revealed that the SUVmax at baseline and DAS28-ESR at 6 months were factors associated with joint destruction at 2 years (odds ratio [OR] 1.350, 95% confidence interval [CI]: 1.013–1.798 and OR 1.954, 95%CI: 1.375–2.779, respectively).

## DISCUSSION

The findings of assessments of radiographic damage in large joints in comparison with that observed in small joints have been reported only in a limited number of studies. However, it is well recognized that RA affects large joints at an incidence of 21–56% during the disease process.^16^ Kuper et al found that, after 6 years of follow-up, 50% of RA patients demonstrated radiographic abnormalities in at least 1 large joint,^7^ and radiographic damage of the large joints in the setting of RA substantially contributes to physical disability, with a negative effect on the quality of life of the patient.^7,8^

Drossaers-Bakker et al reported similar correlations between radiographic damage of the large and small joints associated with disability. The authors found the disease activity and radiographic damage of the large joints to be main contributors to variation in the Health Assessment Questionnaire (HAQ) scores in a multivariate analysis.^8^ In addition, Tanaka et al reported that damage to large joints, especially the shoulder, elbow, and knee, is the major determinant of the RA disease activity, as assessed according to a patient-oriented evaluation of pain using a visual analog scale (VAS), the patient's and physician's general VAS, and the HAQ.^17^ Therefore, it is important to assess the extent of joint destruction of large joints in RA patients.

Over the past decade, new imaging modalities, such as magnetic resonance imaging (MRI) and ultrasonography (US), have been established to assess the degree of synovitis in RA-affected joints. MRI has become an important modality due to its ability to visualize synovial inflammation as contrast-enhanced lesions, especially in the wrist and finger joints. However, RA often affects large joints throughout the entire body, and MRI is currently not effective for assessing multiple distant joints or misfits with metallic implants. US has been reported to be more sensitive and reliable than physical examinations in detecting synovial hypertrophy, effusion, and inflammation.^18^ Although US examinations are cheap and widely available, the results have been shown to be observer-dependent.

In the present study, whole-body ^18^F-FDG PET/CT was performed at baseline and 6 months after the initiation of the biological therapy in patients with RA. Whole-body imaging with FDG-PET appears to be a more objective method of assessment. Furthermore, the SUV is highly correlated with the synovial thickness measured on US and MRI.^9,10^ Kubota et al reported the FDG uptake in large joints to be significantly correlated with the CRP level, and a greater FDG uptake was observed in painful/swollen joints. The authors suggested that FDG-PET can be used to identify joints with active RA inflammation more sensitively than clinical signs/symptoms of RA.^6^ Therefore, we performed ^18^F-FDG PET/CT to assess large joints in RA patients before and after biological therapy in this study.

In the present study, higher levels of SUVmax at baseline and 6 months after the initiation of biological therapy were observed in the 33 large joints in which radiographic progression of joint destruction was detected. Because the ΔSUV represents the changes in the clinical status of the affected joints at 6 months, one of our hypotheses is that a low ΔSUV leads to joint destruction. However, there were no significant differences in the ΔSUV values between the patients with and without joint destruction at 2 years.

On the other hand, the SUVmax values at baseline and 6 months and the DAS28-ESR scores at 6 months in the joint destruction progression group were significantly high in this study. These results suggest that determining the disease activity for the entire body, as well as for a single joint, is therefore important to avoid joint destruction of the large joints within 2 years, especially in RA patients with joints exhibiting high SUVmax levels at the initiation of biological therapy. In other words, RA patients with a high disease activity should be treated as soon as possible, as the continuation of a high disease activity may contribute to further joint destruction. Previous studies also suggest that early intervention aimed at preventing radiographic damage before or at the onset of minor radiographic changes may help to preserve the joint function.^7,19^

There are some limitations associated with this study. First there is concern regarding radiographic assessments performed using the Larsen grade. In this method, various changes are included within one grade. Therefore, the Larsen score discriminates changes poorly in cases in which the changes in radiographic abnormalities are mild. In addition, it may be difficult to distinguish joint destruction caused by RA from secondary osteoarthritis in large joints detected progression of joint damage with the Larsen method. On this point, further research is important in order to evaluate large joints more precisely using other radiographic scoring systems.

Second is the problem with the follow-up term. A previous study reported that 18% of patients had already developed damage in at least 1 large joint (Larsen ≥ 1) at baseline, without a preference for a specific joint. Within 3 years, 40% of the patients and, within 6 years, almost 50% of the patients showed radiographic changes, especially in the hips, knees, and shoulders.^7^ Furthermore, another study demonstrated that only 30% of the studied patients exhibited no radiographic abnormalities of the large joints after 12 years of follow-up.^8^ On the other hand, Seki et al reported that 6% of large joints in patients with RA showed progression of joint damage after 1 year of treatment with TNF-blocking therapy.^19^ In the present study, 12% of the patients developed radiographic changes after 2 years. These results support the idea that 2 years may be too short a period to properly evaluate radiographic changes in large joints, and further long-term observation is thus required.

Third is the difference in the treatments for each patient. Because each patient had been treated with several DMARDs, there was a possibility that these drugs interfered with clinical parameters and imaging data in this study.

In conclusion, we investigated the associations between destruction of the large joints in RA patients and ^18^F-FDG PET/CT findings, the disease activity, and laboratory parameters after biological treatment and identified factors associated with joint destruction at 2 years. In particular, the FDG uptake at baseline and 6 months and the disease activity at 6, 12, and 24 months were significantly higher in the large joints demonstrating radiographic progression of destruction at 2 years after the initiation of biological therapy. Furthermore, the SUVmax at baseline and DAS28-ESR at 6 months after biological treatment are significant factors predicting joint destruction of the large joints at 2 years.
